# Impact of Post-Thaw Enrichment of Primary Human Hepatocytes on Steatosis, Inflammation, and Fibrosis in the TruVivo^®^ System

**DOI:** 10.3390/ph17121624

**Published:** 2024-12-03

**Authors:** Justin J. Odanga, Sharon M. Anderson, Sharon C. Presnell, Edward L. LeCluyse, Jingsong Chen, Jessica R. Weaver

**Affiliations:** 1 Institute of Regenerative Medicine, LifeNet Health, VA Beach, VA 23453, USA; justin_odanga@lifenethealth.org (J.J.O.); sharon_anderson@lifenethealth.org (S.M.A.); sharon_presnell@lifenethealth.org (S.C.P.); jingsong_chen@lifenethealth.org (J.C.); 2 Research and Development, LifeNet Health, Research Triangle Park, NC 27709, USA; edward_lecluyse@lifenethealth.org

**Keywords:** liver, hepatocytes, human, disease, Percoll

## Abstract

**Background**: Liver diseases are a global health concern. Many in vitro liver models utilize cryopreserved primary human hepatocytes (PHHs), which commonly undergo post-thaw processing through colloidal silica gradients to remove debris and enrich for a viable PHH population. Post-thaw processing effects on healthy PHHs are partially understood, but the consequences of applying disease-origin PHHs to post-thaw density gradient separation have not been described. **Methods**: Using the TruVivo^®^ system, diseased, type 2 diabetes mellitus (T2DM), and fibrotic PHHs were cultured for 14 days after initially being subjected to either low-density (permissive) or high-density (selective) gradients using Percoll-based thawing medium. **Results**: Changes in functionality, including albumin and urea secretion and CYP3A4 activity, were measured in diseased, T2DM, and fibrotic PHHs enriched in low Percoll compared to PHHs enriched in high Percoll. Lipogenesis increased in the PHHs enriched in low Percoll. Higher expression of CK18 and TGF-β, two fibrotic markers, and changes in expression of the macrophage markers CD68 and CD163 were also measured. **Conclusions**: The use of Percoll for the enrichment of PHHs post-thaw results in differences in attachment and functionality, along with changes in diseased phenotypes, in the TruVivo^®^ system.

## 1. Introduction

Chronic liver disease is a significant global public health concern, representing a growing cause of mortality and morbidity worldwide [1,2]. While the onset of liver disease is multi-faceted, non-alcoholic fatty liver disease (NAFLD) remains the most common driver [3]. An estimated 38% of the adult population globally is currently afflicted with NAFLD, resulting in a substantial healthcare and economic burden [4]. NAFLD encompasses an inflammatory spectrum ranging from benign simple steatosis to nonalcoholic steatohepatitis (NASH), eventually progressing to fibrosis, liver cirrhosis, and hepatocellular carcinoma (HCC) [5,6]. As the prevalence of NAFLD is projected to steadily increase over the next decades, potentially rising to 55% of the global population by 2040 [7], it is imperative to develop comprehensive liver injury models to better understand disease pathogenesis and to support the design of effective pharmacological therapies. 

While recent technological developments have resulted in greater complexity of in vitro human cell-based platforms to model NAFLD progression, to date these approaches have not been capable of accurately recapitulating the inflammatory processes and cellular interactions leading to a diseased state [8,9]. Traditional two-dimensional (2D) models fail to maintain phenotypic stability over time and may lack the non-parenchymal cells required for NAFLD fibrogenesis [8,10]. Hepatic 3D model systems, including spheroids, organoids, and liver-on-a-chip, provide a more realistic microenvironment to study metabolic disorders, but often come with greater complexity and cost, and technical challenges associated with reproducibility [8,9]. A model system is needed that maintains the longevity and phenotypic stability of diseased characteristics, including lipid accumulation and inflammation. The early stages of NAFLD, often associated with obesity, type 2 diabetes mellitus (T2DM), and hypertension, are marked by the accumulation of lipids in the liver. Fat accumulation, caused by excess dietary fats, release of free fatty acids (FFAs) from adipose tissue, lipogenesis, or insufficient fatty acid oxidation [6], can result in insulin resistance and steatosis. Once simple steatosis occurs, a cascade of lipotoxicity and activation of immune cells is initiated, resulting initially in inflammation and liver injury, and eventually progressing to fibrosis. Ideally, an in vitro liver disease model should comprise features from all stages of NAFLD.

Animal disease models, primarily murine-based, are widely utilized to study the metabolic pathways and factors underlying NAFLD development and progression. While these models can incorporate genetic and dietary triggers for disease onset, they often do not accurately reproduce human disease due to the use of artificial induction regimes [8,11]. They also fail to fully capture the distinct features of NAFLD. Some animal disease models only display metabolic disease and steatosis, but not liver inflammation and fibrosis, or vice versa. Interspecies differences from preclinical findings are often unsuccessful at reliably predicting human-relevant pathogenesis [8]. Due to the genetic variation in these animal models, they may fail to replicate human etiology. The limitations of existing animal models further highlight the need for improved human liver model systems that maintain hepatic cells from diseased tissues and better retain key elements of human NAFLD. 

TruVivo^®^, an all-human cell-based triculture system, was developed to provide a robust 2D in vitro system that better mimics the in vivo hepatic microenvironment [12,13]. Comprised of three different human cell types, including cryopreserved primary human feeder cells (FCs), consisting of stromal cells and endothelial cells, and cryopreserved primary human hepatocytes (PHHs), this system maintains stable albumin and urea production and Cytochrome P450 (CYP) 3A4 activity for at least two weeks [12,13]. This platform has been further extended to incorporate PHHs selected from tissue donors with a known medical history of NAFLD to generate a diseased TruVivo^®^ system. PHHs derived from diseased liver tissues sustain a characteristic fatty liver disease phenotype for extended culture periods, including decreased hepatocyte functionality, significant basal lipid accumulation, altered lipogenic responses, and increased pro-inflammatory cytokine production [14]. In addition, the diseased TruVivo^®^ system can be modulated to support the investigation of early-stage steatosis development, as well as later-stage disease progression. The presence and longevity of these disease-relevant attributes suggest that the diseased TruVivo^®^ system is a promising tool for advancement of models across the spectrum of liver disease.

To best predict toxicology and safety as a preclinical hepatic system, an improved understanding of how the diseased PHH phenotype manifests under varying culture conditions is needed. In these studies, PHHs in the TruVivo^®^ system were cultured following post-thaw processing in low and high-percentage Percoll-based thawing medium. Percoll colloidal silica medium is well-established for density-based cell separation, including epithelial and fibroblastic cells, allowing for separation and/or enrichment of unique cell populations [15]. In low-density Percoll conditions, diseased PHHs were found to have lower attachment and increased fat accumulation on day 14 of culture as compared to higher-Percoll thawing medium, while hepatic functions of albumin and urea production and CYP3A4 activity were also impacted. Expression of the fibrotic markers cytokeratin-18 (CK18) and transforming growth factor beta (TGF-β) and the macrophage markers CD68 and CD163 were also highest when diseased PHHs were enriched in low versus high Percoll, as was expression of genes related to production of enzymes that are critical to gluconeogenesis. In a subset of PHHs derived from T2DM donors, expression of fibrotic markers was altered following low or high Percoll enrichment. Taken together, treatment with either a low or high Percoll thawing medium can support a sustained diseased phenotype with differences in attachment, functionality, lipogenesis, and marker expression in the TruVivo^®^ system. 

## 2. Results

### 2.1. Characterization of T2DM Donors After Enrichment in Low and High Percoll

**Figure 1:**
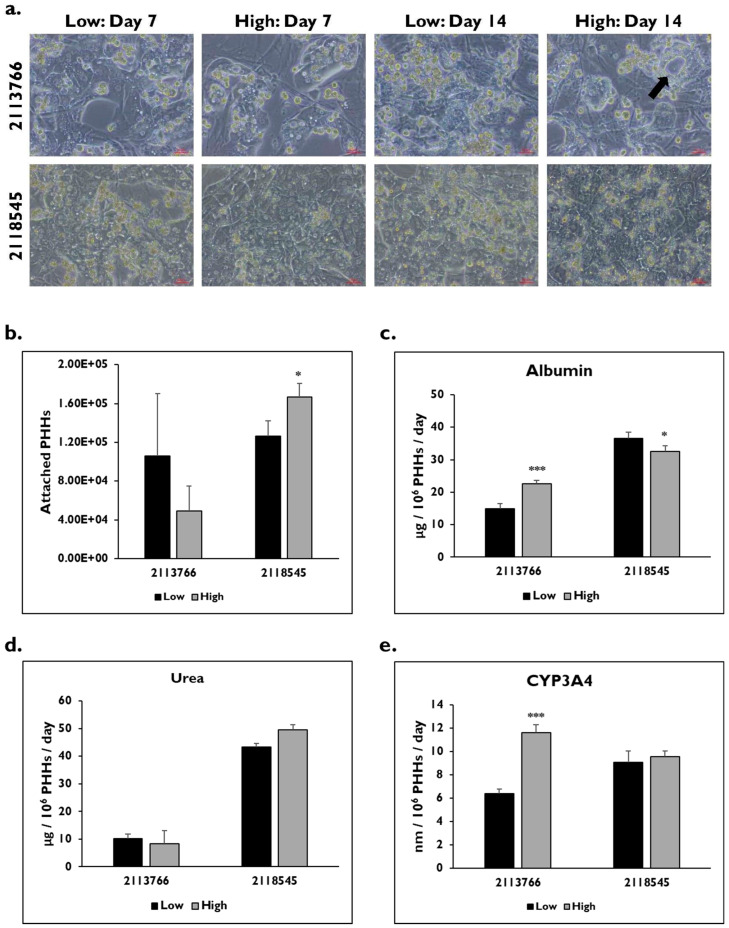
PHHs from Type 2 diabetes mellitus (T2DM) donors enriched in either low or high Percoll show differences in morphology and function. (**a**) Morphology of T2DM lots 2113766 (top row) and 2118545 (bottom row) on days 7 and 14 after enrichment in low and high Percoll. Arrow indicates vacuole. Magnification = 20×. Scale bar = 50 μm. (**b**) Attached number of PHHs on day 14 after enrichment in low (black bars) and high (grey bars) Percoll. n = 5 images per condition for each donor lot. (**c**) Albumin and (**d**) urea levels normalized to number of attached PHHs on day 14 after enrichment in low (black bars) and high (grey bars) Percoll. n ≥ 2 samples per condition. (**e**) CYP3A4 activity on day 14 from PHHs enriched in low (black bars) and high (grey bars) Percoll. n ≥ 3 samples per condition. Error bars represent standard deviation. * *p* ≤ 0.05 and *** *p* ≤ 0.001 to low Percoll.

Two T2DM donors, 2113766 and 2118545, were enriched in low and high Percoll after thawing (Figure 1). Although these donors did not have a NAS score high enough to classify them as diseased, T2DM is strongly correlated with NAFLD [16,17]. There were distinct morphological differences on days 7 and 14 between these donor lots after enrichment in both thawing media (Figure 1a). Donor lot 2118545 had the normal cuboidal cell shape and formed hepatocyte colonies. Irregularly shaped hepatocytes with large vacuoles were seen in donor lot 2113766. Although there were differences in attachment after the use of different thawing media for donor lot 2113766 (105,856 ± 64,145 vs. 49,472 ± 15,970 PHHs), they were not significant (*p* = 0.129) (Figure 1b). Enrichment in the low Percoll thawing medium resulted in significantly lower attachment (*p* = 0.021) for donor lot 2118545 compared to the high Percoll thawing medium (126,304 ± 25,583 vs. 166,848 ± 14,063 PHHs).

Differences in functionality were measured within the donor lots after enrichment using either thawing medium. For donor lot 2113766, significant differences were seen in albumin levels (low: 15.0 ± 1.4 vs. high: 22.6 ± 2.0 μg/10^6^ PHHs/day) (*p* = 0.000) (Figure 1c), but not in urea levels (low: 10.1 ± 1.8 vs. high: 8.4 ± 1.4 μg/10^6^ PHHs/day) (*p* = 0.099) (Figure 1d), on day 14. Baseline CYP3A4 levels on day 14 were significantly lower in the PHHs enriched in the low Percoll (6.4 ± 0.4 nm/10^6^ PHHs/day) versus those enriched in high Percoll (11.6 ± 0.7 nm/10^6^ PHHs/day) (*p* = 0.000) (Figure 1e). Significantly higher levels of albumin were seen in donor lot 2118545 after enrichment in low Percoll compared to high Percoll (low: 36.5 ± 1.0 vs. high: 32.6 ± 1.7 µg/10^6^ PHHs/day) (*p* = 0.018). There were no significant differences seen in urea levels (low: 43.3 ± 4.7 vs. high: 49.5 ± 1.8 µg/10^6^ PHHs/day) (*p* = 0.090) and baseline CYP3A4 activity (low: 9.1 ± 1.0 vs. high: 9.6 ± 0.4 nm/10^6^ PHHs/day) (*p* = 0.274) after post-thaw enrichment in either low or high Percoll.

**Figure 2:**
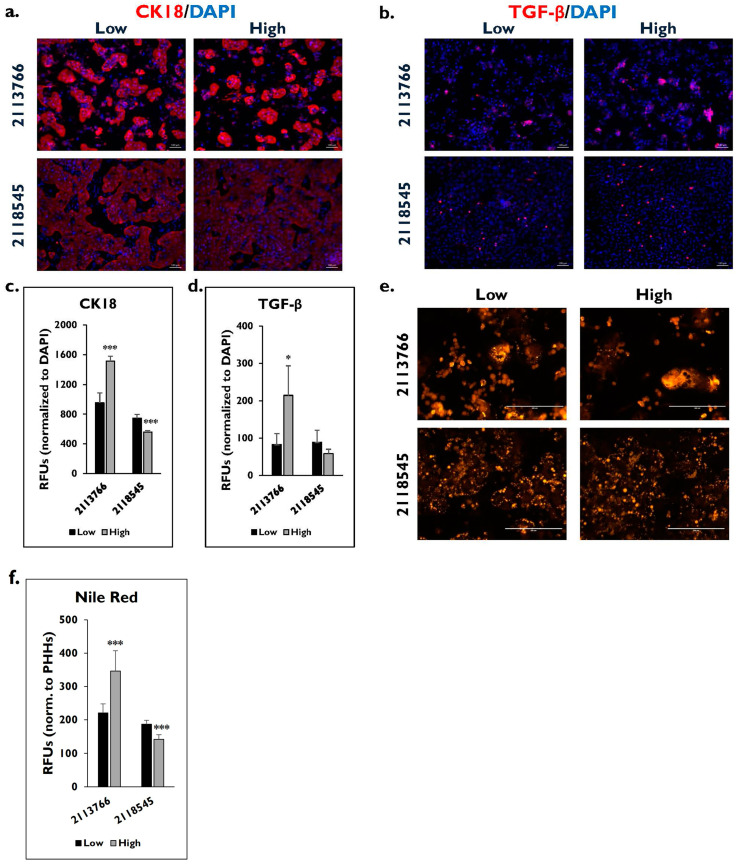
Differences in fibrotic marker expression were measured from low and high Percoll-enriched PHHs from Type 2 diabetes mellitus (T2DM) donors. Marker expression of fibrotic proteins (**a**) CK18 (red) and (**b**) TGF-β (red) plus DAPI staining (blue) on day 14 in T2DM lots 2113766 (top row) and 2118545 (bottom row) after enrichment in low (left column) and high (right column) Percoll. Magnification = 10×. Scale bar = 100 μm. Quantification of (**c**) CK18 and (**d**) TGF-β marker expression in PHHs after enrichment in low (black bars) and high (grey bars) Percoll. n = 5 images per condition for each donor. (**e**) Nile red staining of T2DM lots 2113766 (top row) and 2118545 (bottom row) on day 14 after enrichment in low (left column) and high Percoll (right column). Magnification = 20×. Scale bar = 200 μm. (**f**) Quantification of fluorescence from Nile Red staining in relative fluorescent units (RFUs) after PHHs have been enriched in low (black bars) and high (grey bars) Percoll. n ≥ 5 images per condition for each donor lot. Values have been normalized to attached number of PHHs for each donor lot and condition. Error bars represent standard deviation. * *p* ≤ 0.05 and *** *p* ≤ 0.001 to low Percoll.

Donor lot 2113766 had a fibrosis score of 2 compared to a fibrosis score of 0 for donor lot 2118545. The fibrotic markers, CK18 and TGF-β, were examined to determine differences in expression between the cell populations enriched in either low or high Percoll (Figure 2). Representative images of CK18 (Figure 2a) and TGF-β (Figure 2b) on day 14 showed more intense staining for both markers in donor lot 2113766 after enrichment in either low or high Percoll. After quantitating the fluorescent signal, expression was significantly higher in PHHs enriched in high Percoll for CK18 (low: 953.4 ± 133.8 vs. high: 1513 ± 67.5 RFUs/PHHs) (*p* = 0.000) (Figure 2c) and TGF-β (low: 82.7 ± 29.3 vs. high: 214.2 ± 79 RFUs/PHHs) (*p* = 0.018) (Figure 2d) for donor lot 2113766. Fibrotic marker expression for CK18 was significantly higher in the low Percoll-enriched PHHs (747 ± 51.3 RFUs/PHHs) compared to the PHHs enriched in high Percoll (559 ± 17.4 RFUs/PHHs) (*p* = 0.001) for donor lot 2118545. Although TGF-β was higher in the low enriched Percoll PHHs (89.2 ± 31.4 RFUs/PHHs), it was not significant compared to the PHHs enriched in the high Percoll (58.4 ± 11.6 RFUs/PHHs) (*p* = 0.094).

Lipogenesis on day 14 from these two lots was examined using Nile Red staining between PHHs that were enriched in either low or high Percoll (Figure 2e). Following enrichment with either low or high Percoll, lot 2113766 appeared to have more intense staining throughout the PHHs as compared to lot 2118545, where staining was less intense and concentrated on the periphery. When the fluorescence was quantitated, PHHs from lot 2113766 enriched in high Percoll had significantly higher fluorescence (346.4 ± 61.1 RFUs) compared to those enriched in low Percoll (low: 221.1 ± 27.1 RFUs) (*p* = 0.000) (Figure 2f). The opposite was measured in PHH lot 2118545. A significantly higher fluorescent signal was measured from PHHs enriched in the low Percoll (187.3 ± 11.6 RFUs) than those enriched in the high Percoll (142.3 ± 13.1 RFUs) (*p* = 0.001). 

### 2.2. Characterization of Diseased Steatotic Donors After Enrichment in Low and High Percoll

**Figure 3:**
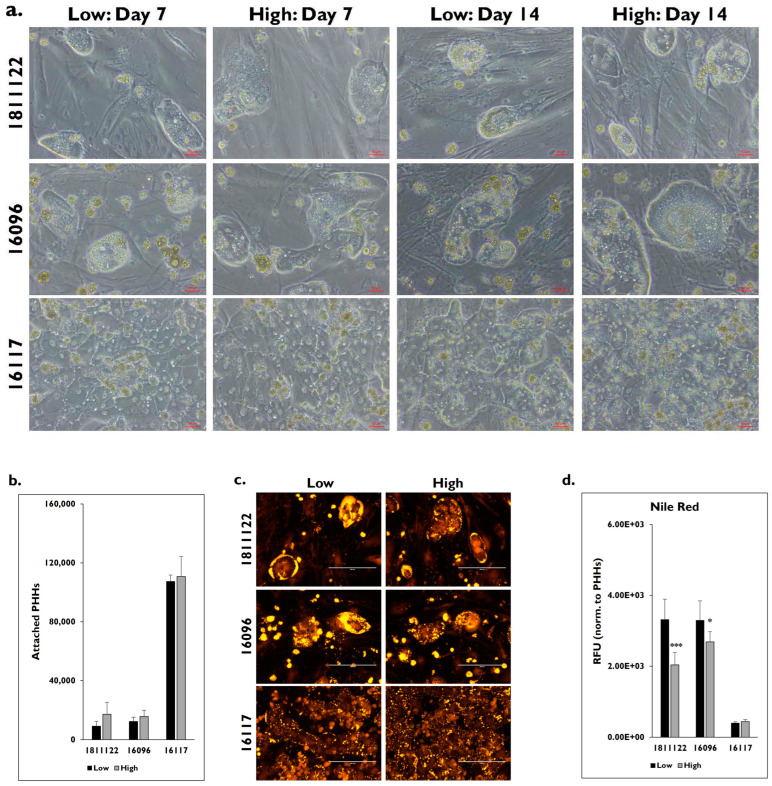
Diseased PHHs enriched in low Percoll have increased lipid accumulation compared to diseased PHHs enriched in high Percoll. (**a**) Morphology of diseased donor lots 1811122 (top row) and 16096 (middle row) and normal donor lot 16117 (bottom row) on days 7 and 14 after enrichment in low and high Percoll. Magnification = 20×. Scale bar = 50 μm. (**b**) Number of attached PHHs on day 14 after enrichment in low (black bars) and high (grey bars) Percoll. n = 5 images per condition for each donor lot. (**c**) Staining of lipids by Nile Red in diseased lots 1811122 (top row) and 16096 (middle row) and normal lot 16117 (bottom row) on day 14. PHHs were enriched in low (left column) and high Percoll (right column). Magnification = 20×. Scale bar = 200 μm. (**d**) Quantification of fluorescence from Nile Red staining in Relative Fluorescent Units (RFUs) after enrichment in low (black bars) and high (grey bars) Percoll. n ≥ 5 images per condition for each donor lot. Values are normalized to number of attached PHHs for each lot and condition. Error bars represent standard deviation. * *p* ≤ 0.05 and *** *p* ≤ 0.001 to low Percoll.

Two diseased donor lots, 1811122 and 16096, and one normal donor lot, 16117, were thawed and then enriched using either low or high Percoll (Figure 3). Donor lot 1811122 has a steatosis score of 3, while a score of 2 was given to donor lot 16096. Normal donor lot 16117 has a steatosis score of 0. There were morphological differences on days 7 and 14 in the diseased donor lots that were not seen in the normal donor lot after enrichment in either the low or high Percoll (Figure 3a). No significant differences in attachment were determined for donor lots 1811122 (low: 9120 ± 3194 vs. high: 17,120 ± 8102 PHHs) (*p* = 0.159), 16096 (low: 12,352 ± 2838 vs. high: 15,648 ± 4212 PHHs) (*p* = 0.190), and 16117 (low: 107,360 ± 4302 vs. high: 110,656 ± 13,541 PHHs) (*p* = 0.636) after enrichment using either thawing medium (Figure 3b). However, PHHs from normal donor lot 16117 had significantly higher attachment after enrichment in either low or high Percoll compared to the PHHs enriched in either low or high Percoll from the diseased donor lots. 

When steatosis was examined, the diseased PHHs appeared to have increased lipogenesis compared to the normal lot when stained using Nile Red on day 14 (Figure 3c). The diseased lots enriched in the low Percoll appeared to have more intense staining throughout the hepatocytes and therefore more lipid accumulation compared to the diseased PHHs enriched in the high Percoll. Lipid accumulation was seen along the periphery in the normal donor lot and was less intense compared to the diseased lots. When Nile Red staining was quantified, the diseased lots had significantly higher fluorescence after enrichment in the low Percoll (1811122: 3312 ± 580 RFUs; 16096: 3288 ± 553 RFUs) compared to those enriched in the high Percoll (1811122: 2035 ± 352 RFUs; 16096: 2684 ± 292 RFUs) (1811122 *p* = 0.000; 16096 *p* = 0.016) (Figure 3d). There was no significant difference between the fluorescent signal from the normal donor lot 161117 after enrichment in either the low (395.6 ± 35 RFUs) or high Percoll (436.7 ± 60.1 RFUs) (*p* = 0.175).

**Figure 4:**
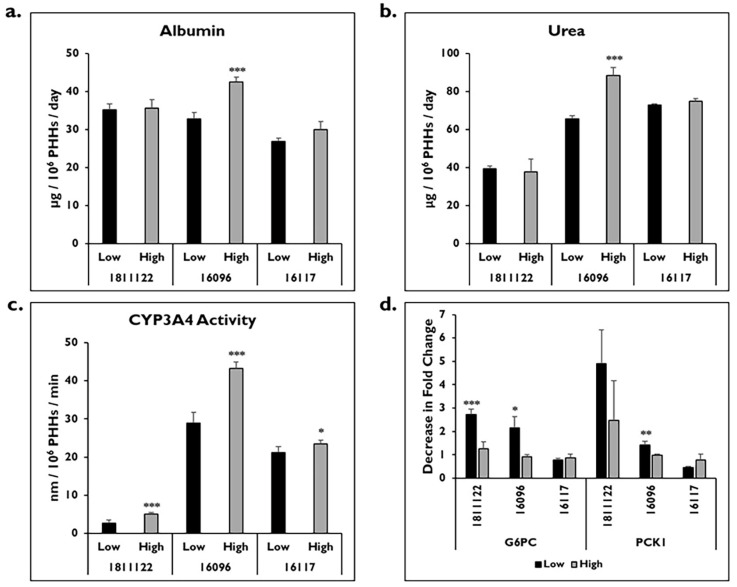
CYP3A4 activity decreases in diseased lots enriched in low Percoll. (**a**) Albumin and (**b**) urea levels on day 14 normalized to attached PHHs in diseased lots 1811122 and 16096, and normal lot 16117 enriched in low (black bars) and high (grey bars) Percoll. n ≥ 2 samples per condition. *** *p* < 0.001 to low Percoll. (**c**) Baseline CYP3A4 activity on day 14 normalized to attached PHHs after enrichment in low (black bars) and high (grey bars) Percoll. n ≥ 3 samples per condition * *p* < 0.05 and *** *p* < 0.001 to low Percoll. (**d**) *G6PC* and *PCK1* gene expression represented as decrease in fold change on day 14 in lots enriched in low (black bars) and high (grey bars) Percoll. n ≥ 2 samples per condition. Data normalized to PHHs enriched in high Percoll. Error bars represent standard deviation. * *p* ≤ 0.05, ** *p* ≤ 0.01, and *** *p* ≤ 0.001 to high Percoll.

The functionality of the diseased donor lots and the normal donor lot was determined, including albumin and urea levels and CYP3A4 activity on day 14 (Figure 4). There were no significant differences in albumin levels after enrichment in low and high Percoll for diseased lot 1811122 (low: 35.2 ± 1.6 vs. high: 35.6 ± 2.2 μg/10^6^ PHHs/day) (*p* = 0.775) and normal lot 16117 (low: 26.9 ± 0.9 vs. high: 29.9 ± 2.1 μg/10^6^ PHHs/day) (*p* = 0.053) (Figure 4a). Significantly higher levels of albumin were measured from diseased donor lot 16096 after enrichment in high Percoll (42.5 ± 1.3 μg/10^6^ PHHs/day) compared to low Percoll (32.8 ± 1.7 μg/10^6^ PHHs/day) (*p* = 0.000). PHHs from this donor lot enriched in high Percoll also had significantly higher levels of urea than those enriched in low Percoll (low: 65.7 ± 1.7 vs. high: 88.5 ± 4.1 μg/10^6^ PHHs/day) (*p* = 0.000) (Figure 4b). There were no significant differences in urea levels in donor lots 18111122 and 16117 after enrichment in either low (1811122: 39.3 ± 1.5 μg/10^6^ PHHs/day; 16117: 72.8 ± 0.6 μg/10^6^ PHHs/day) or high Percoll (1811122: 37.8 ± 6.8 μg/10^6^ PHHs/day; 16117: 74.8 ± 1.5 μg/10^6^ PHHs/day) (1811122 *p* = 0.685; 16117 *p* = 0.096).

In addition to albumin and urea, baseline CYP3A4 activity was measured for all three donor lots and was significantly higher in PHHs enriched in high Percoll (Figure 4c). Diseased donor lot 16096 had the highest activity (low: 28.9 ± 2.7 vs. high: 43.3 ± 1.7 nm/10^6^ PHHs/min) (*p* = 0.000), while the other diseased donor lot 1811122 had the lowest activity (low: 2.7 ± 0.7 vs. high: 5.0 ± 0.5 nm/10^6^ PHHs/min) (*p* = 0.000). Donor lot 16117 had significantly lower CYP activity in PHHs enriched in low Percoll (21.2 ± 1.5 nm/10^6^ PHHs/min) compared to those PHHs enriched in high Percoll (23.4 ± 1.1 nm/10^6^ PHHs/min) (*p* = 0.019). 

Gene expression of *glucose-6-phosphatase catalytic subunit* (*G6PC*) and *phosphoenolpyruvate carboxykinase 1* (*PCK1*) were measured on day 14 in these donor lots after enrichment in low and high Percoll (Figure 4d). Expression of the housekeeping gene *glyceraldehyde 3-phosphate dehydrogenase* (*GAPDH*) was stable across the different donors under the various conditions. There was a significant fold-change decrease in *G6PC* gene expression after enrichment in the low Percoll for both diseased lots 1811122 (2.7 ± 0.2) and 16096 (2.2 ± 0.5) when normalized to the PHHs enriched in the high Percoll (1811122: 1.3 ± 0.3; 16096: 0.9 ± 0.1) (1811122 *p* = 0.000; 16096 *p* = 0.015). No change in expression of this gene was measured after enrichment in low Percoll (0.8 ± 0.1) when normalized to PHHs enriched in high Percoll (0.9 ± 0.2) (*p* = 0.415) for normal donor lot 16117. For *PCK1* gene expression, PHHs from diseased lot 16096 enriched in low Percoll (1.4 ± 0.2) had significantly decreased fold-change expression when normalized to PHHs enriched in high Percoll (1.0 ± 0.1) (*p* = 0.005). Although PHHs from diseased lot 1811122 enriched in low Percoll had decreased gene expression (4.9 ± 1.4) after normalization to high Percoll-enriched PHHs (2.5 ± 1.7), it was not significant (*p* = 0.080). No differences in *PCK1* gene expression were seen in PHHs enriched in low Percoll (0.5 ± 0.0) when normalized to PHHs enriched in high Percoll (0.8 ± 0.3) from normal donor lot 16117 (*p* = 0.088). 

### 2.3. Differences in Expression of Fibrotic Markers After Enrichment of PHHs in Low and High Percoll

**Figure 5:**
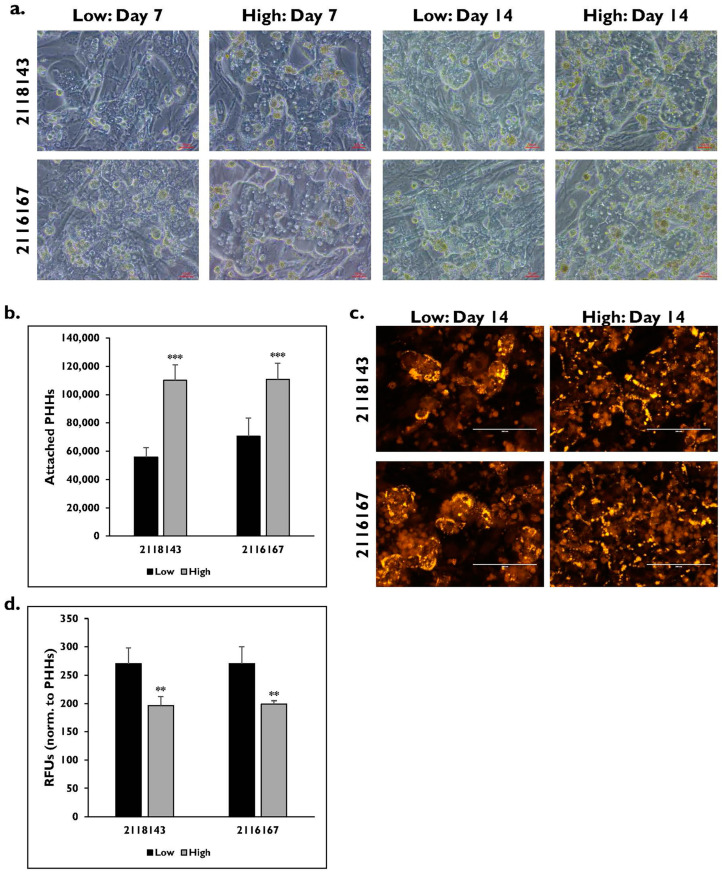
Fibrotic PHHs have lower attachment after enrichment in low Percoll compared to fibrotic PHHs enriched in high Percoll. (**a**) Morphology of fibrotic lots 2118143 (top row) and 2116167 (bottom row) on days 7 and 14 after enrichment in low and high Percoll. Magnification = 20×. Scale bar = 50 μm. (**b**) Number of attached PHHs on day 14 after enrichment in low (black bars) and high (grey bars) Percoll. n = 5 images per condition for each donor lot. (**c**) Nile Red staining on hepatocyte lots 2118143 (top row) and 2116167 (bottom row) on day 14 in PHHs enriched in low and high Percoll. Magnification = 20×. Scale bar = 200 μm. (**d**) Quantification of fluorescence in relative fluorescent units (RFUs) from Nile Red staining when PHHs were enriched in low (black bars) and high (grey bars) Percoll. n ≥ 3 images per condition for each donor lot. Values have been normalized to number of attached PHHs for each lot and condition. Error bars represent standard deviation. ** *p* ≤ 0.01 and *** *p* ≤ 0.001 to low Percoll.

Two additional donor lots, 2118143 and 2116167, with fibrosis scores of 2 and 1 and a normal donor lot, 16117, with a fibrosis score of 0, were enriched in low and high Percoll after thawing (Figure 5). The PHHs in the fibrotic donor lots enriched in the low Percoll appeared to have a different morphology on days 7 and 14 compared to the PHHs enriched in the high Percoll (Figure 5a). These high Percoll-enriched PHHs were cuboidal in shape, multinucleated, and formed hepatic colonies with well-defined borders by day 7. This morphology was maintained throughout the 14-day culture period. However, the low Percoll-enriched PHHs appeared flat with less distinct colony formation and borders. Differences in attachment on day 14 were measured after enrichment using different thawing media (Figure 5b). The PHHs enriched in the low Percoll attached significantly less compared to the high Percoll-enriched PHHs for the fibrotic lots 2118143 (low: 55,856 ± 6624 vs. high: 110,092 ± 11,079 PHHs) (2118143 *p* = 0.000) and 2116167 (low: 70,502 ± 12,866 vs. high: 110,783 ± 11,288 PHHs) (2116167 *p* = 0.000). 

Differences in lipogenesis on day 14 were determined after enrichment in low and high Percoll for lots 2118143 and 2116167 (Figure 5c). Similar Nile Red staining patterns were seen in both lots with lipids accumulating along the cell periphery and within the PHHs enriched using the low Percoll. PHHs enriched in high Percoll appeared to have lipids located mostly along the periphery and little to no lipid accumulation within the cells. Quantification of Nile Red fluorescent signal was determined (Figure 5d). There was significantly higher fluorescent signal measured in the PHHs enriched in the low Percoll (2118143: 270.0 ± 28.1 RFUs; 2116167: 270.0 ± 30.3 RFUs) compared to those enriched in the high Percoll (2118143: 195.9 ± 16.0 RFUs; 2116167: 198.8 ± 6.0 RFUs) (2118143 *p* = 0.002; 2116167 *p* = 0.007). 

**Figure 6:**
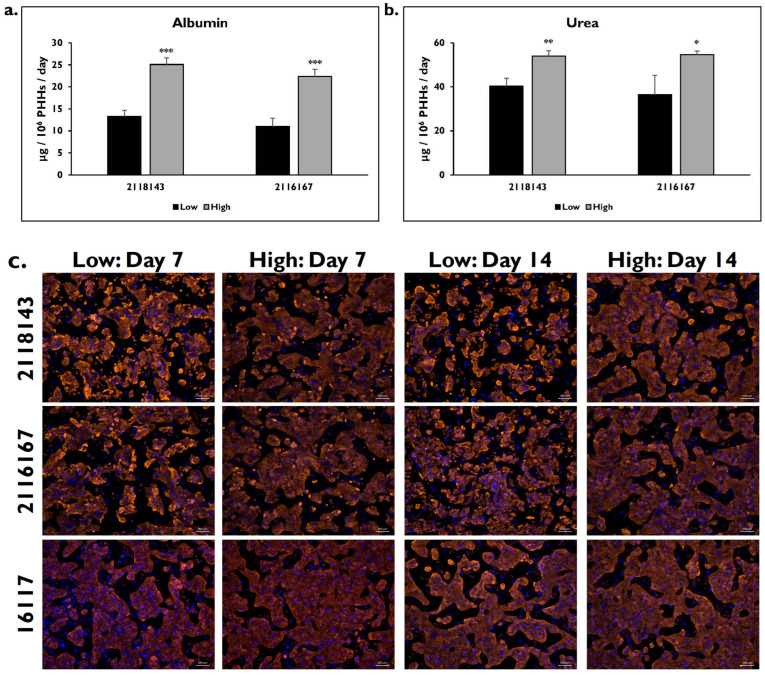

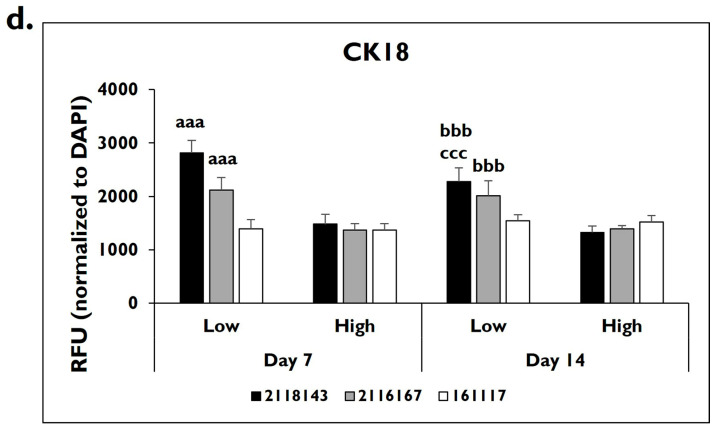
Cytokeratin-18 expression is higher in the fibrotic lots enriched in low Percoll. (**a**) Albumin and (**b**) urea levels on day 14 from diseased lots 2118143 and 2116167 after enrichment in low (black bars) and high (grey bars) Percoll. n ≥ 2 samples per condition. * *p* ≤ 0.05, ** *p* ≤ 0.01, and *** *p* ≤ 0.001 to low Percoll. (**c**) CK18 protein expression (red) plus DAPI (blue) on days 7 and 14 in lots 2118143 (top row), 2116167 (middle row), and 16117 (bottom row) after enrichment in low and high Percoll. Magnification = 10×. Scale bar = 100 μm. (**d**) CK18 quantification in relative fluorescent units (RFU) normalized to DAPI number on days 7 and 14 in lots 2118143 (black bars), 2116167 (grey bars), and 16117 (white bars) after enrichment in low and high Percoll. n = 5 images per condition for each donor lot. Error bars represent standard deviation. ^aaa^ *p* ≤ 0.001 to high Percoll on day 7. ^bbb^ *p* ≤ 0.001 to high Percoll on day 14. ^ccc^ *p* ≤ 0.001 to low Percoll on day 7.

Hepatocyte function was determined for each lot after being enriched in either the low or high Percoll (Figure 6). There were significantly lower levels of albumin for lots 2118143 and 2116167 after enrichment in low (2118143: 13.3 ± 1.4 μg/10^6^ PHHs/day; 2116167: 11.0 ± 1.9 μg/10^6^ PHHs/day) versus high (2118143: 25.1 ± 1.5 μg/10^6^ PHHs/day; 2116167: 22.4 ± 1.7 μg/10^6^ PHHs/day) Percoll (2118143 *p* = 0.000; 2116167 *p* = 0.000) (Figure 6a). The level of urea was also significantly higher in the PHHs enriched using the high Percoll for both lots (2118143: 53.9 ± 2.5 μg/10^6^ PHHs/day; 2116167: 54.7 ± 1.7 μg/10^6^ PHHs/day) compared to PHHs enriched in low Percoll (2118143: 40.2 ± 3.7 μg/10^6^ PHHs/day; 2116167: 36.5 ± 8.7 μg/10^6^ PHHs/day) (2118143 *p* = 0.002; 2116167 *p* = 0.027) (Figure 6b). 

Expression of the fibrosis marker CK18 on days 7 and 14 was determined in each lot after enrichment using the two different Percoll media (Figure 6c). CK18 staining appeared to be more intense in the fibrotic lots 2118143 and 2116167 at both time points after enrichment in the low Percoll. Quantification of this staining on day 7 showed significantly higher levels of fluorescence in the PHHs enriched in the low Percoll for lots 2118143 (2812.6 ± 235.3 RFUs) and 2116167 (2115.9 ± 234.2 RFUs) compared to those enriched in the high Percoll (2118143: 1479.2 ± 183.8 RFUs; 2116167: 1373.1 ± 117.9 RFUs) (2118143 *p* = 0.000; 2116167 *p* = 0.000) (Figure 6d). Significantly higher staining was also determined on day 14 for both lots when enriched in low Percoll (2118143: 2279.7 ± 257.1 RFUs; 2116167: 2009.3 ± 286.9 RFUs) compared to high Percoll (2118143: 1324.4 ± 118.4; 2116167: 1390.8 ± 63.9 RFUs) (2118143 *p* = 0.000; 2116167 *p* = 0.000). For lot 2118143, a significant decrease in CK18 staining was measured on day 14 compared to day 7 (*p* = 0.000) from the PHHs enriched in the low Percoll. This decrease was not seen in the other lots. There were no significant differences seen after enrichment in either low (day 7: 1391.9 ± 174.5 RFUs; day 14: 1544.4 ± 109.9 RFUs) or high Percoll (day 7: 1367.5 ± 123.5 RFUs; day 14: 1518.1 ± 125.7 RFUs) (day 7 *p* = 0.978; day 14 *p* = 0.972) for the normal lot 16117. 

### 2.4. Inflammation in Fibrotic PHHs After Enrichment in Low and High Percoll

**Figure 7:**
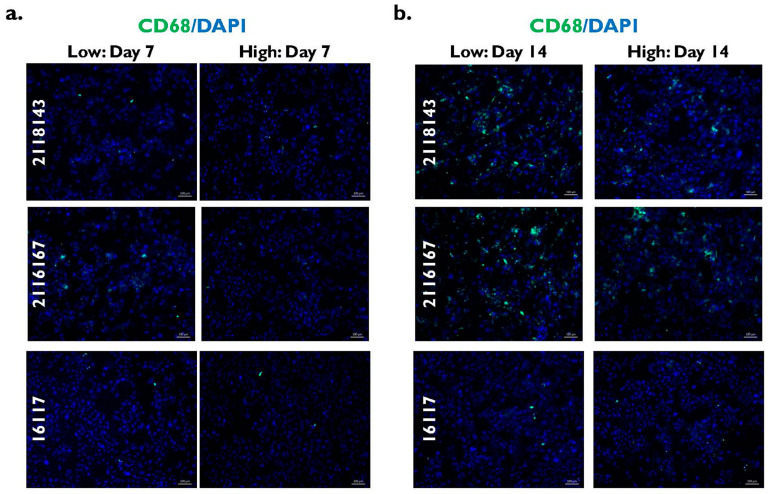

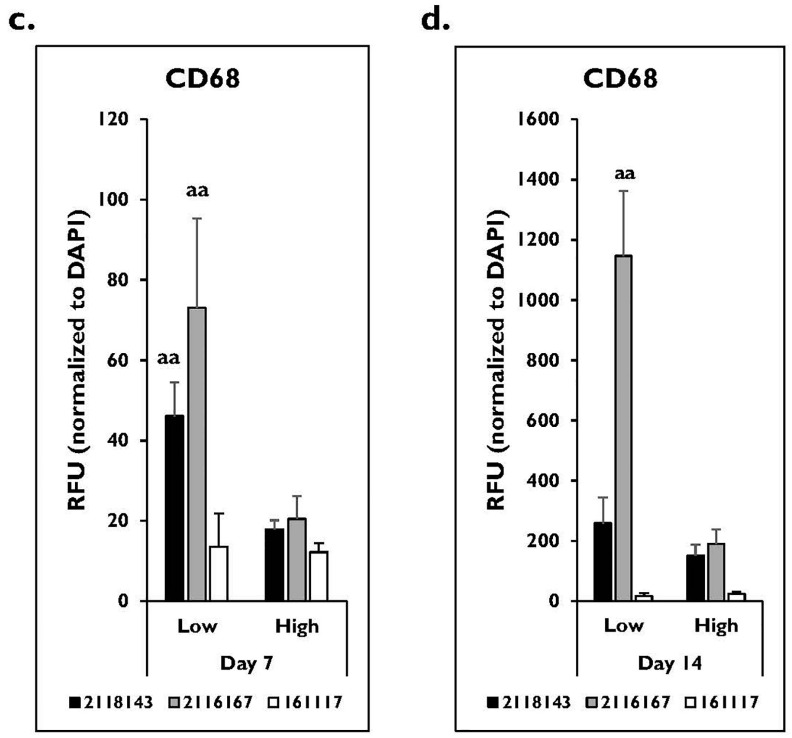
CD68 marker expression is higher in fibrotic PHHs enriched in low Percoll versus fibrotic PHHs enriched in high Percoll on days 7 and 14. CD68 (green) marker expression plus DAPI (blue) on (**a**) days 7 and (**b**) 14 in lots 2118143 (top row), 2116167 (middle row) and 16117 (bottom row) after being enriched in low and high Percoll. Magnification = 10×. Scale bar = 100 μm. CD68 quantification in Relative Fluorescent Units (RFU) normalized to DAPI number on (**c**) days 7 and (**d**) 14 in lots 2118143 (black bars), 2116167 (grey bars), and 16117 (white bars) after enrichment in low and high Percoll. n = 5 images per condition for each donor lot. Error bars represent standard deviation. ^aa^ *p* ≤ 0.01 to high Percoll.

When staining for the macrophage marker CD68 was performed in the fibrotic donor lots 2118143 and 2116167 (Figure 7), there was light staining on day 7 in PHHs enriched in low Percoll compared to those PHHs enriched in high Percoll (Figure 7a). More intense staining was seen on day 14 in the fibrotic lots compared to day 7 (Figure 7b). The fibrotic lot 2116167 appeared to have the most intense CD68 staining on day 14 from PHHs enriched in either Percoll thawing medium. There appeared to be little to no change in staining intensity on days 7 and 14 in the normal donor lot 16117 from PHHs enriched in either low or high Percoll. When the fluorescent signal was quantified, the signal on day 7 was significantly higher in the fibrotic donor PHHs enriched in the low Percoll (2118143: 46.1 ± 8.4 RFUs; 2116167: 73.0 ± 22.3 RFUs) compared to those PHHs enriched in the high Percoll (2118143: 17.8 ± 2.3 RFUs; 2116167: 20.5 ± 5.6 RFUs) (2118143 *p* = 0.002; 2116167 *p* = 0.007) (Figure 7c). It was also higher in the PHHs enriched in the low Percoll for both fibrotic lots 2118143 (258.1 ± 85.8 RFUs) and 2116167 (1146.4 ± 216.6 RFUs) compared to PHHs enriched in the high Percoll (2118143: 151.9 ± 35.9 RFUs; 2116167: 189.9 ± 48.2 RFUs) on day 14 (2118143 *p* = 0.103; 2116167 *p* = 0.003) (Figure 7d). However, only fibrotic lot 2116167 had a significantly higher signal. No significant differences were determined in the normal lot 16117 on days 7 (low 13.6 ± 8.4 vs. high 12.2 ± 2.3 RFUs) (*p* = 0.742) and 14 (low 16.8 ± 9.3 vs. high 24.1 ± 7.5 RFUs) (*p* = 0.211) using either Percoll thawing medium.

**Figure 8:**
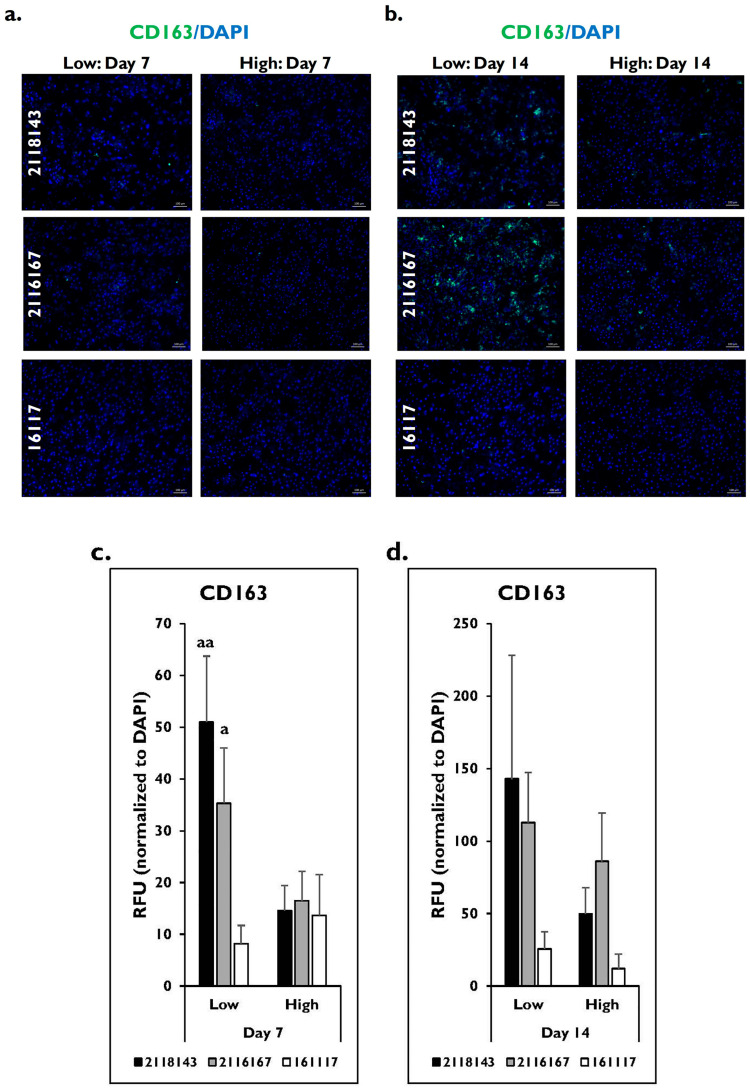
Fibrotic PHHs enriched in low Percoll have increased CD163 marker expression compared to those enriched in high Percoll on day 7 but not day 14. CD163 (green) marker expression plus DAPI (blue) on (**a**) days 7 and (**b**) 14 in lots 2118143 (top row), 2116167 (middle row) and 16117 (bottom row) after being enriched in low and high Percoll. Magnification = 10×. Scale bar = 100 μm. CD163 quantification in relative fluorescent units (RFU) normalized to DAPI number on (**c**) days 7 and (**d**) 14 in lots 2118143 (black bars), 2116167 (grey bars), and 16117 (white bars) after enrichment in low and high Percoll. n = 5 images per condition for each donor lot. Error bars represent standard deviation. ^a^ *p* ≤ 0.05 and ^aa^ *p* ≤ 0.01 to high Percoll.

A similar pattern of expression was seen for the macrophage marker CD163 in the fibrotic versus normal donor lots (Figure 8). Little to no fluorescence was seen in the PHHs on day 7 from any of the three lots after enrichment in either low or high Percoll (Figure 8a). There appeared to be an increase in fluorescent intensity on day 14 in the fibrotic lots from the PHHs enriched in the low and high Percoll (Figure 8b). This intensity change was not visually seen in the normal donor lot after enrichment in either Percoll thawing medium. Fluorescent signal was quantified for CD163 expression. The signal on day 7 was significantly higher in the low Percoll-enriched PHHs (2118143 low: 50.9 ± 12.7 RFUs; 2116167 low: 35.3 ± 10.7 RFUs) compared to the high Percoll-enriched PHHs (2118143 high: 14.6 ± 4.8 RFUs; 2116167 high: 16.5 ± 5.7 RFUs) (2118143 *p* = 0.002; 2116167 *p* = 0.013) (Figure 8c). A higher level of marker expression was measured in the fibrotic lots 2118143 and 2116167 on day 14 in PHHs enriched in low Percoll (2118143 low: 142.9 ± 85.2 RFUs; 2116167 low: 112.9 ± 34.5 RFUs) versus high Percoll (2118143 high: 49.8 ± 18.1 RFUs; 2116167 high: 86.1 ± 33.2 RFUs) (2118143 *p* = 0.075; 2116167 *p* = 0.284) (Figure 8d). However, it was not significantly different for any donor. No significant differences were determined in the normal lot 16117 on days 7 (low 8.1 ± 3.6 vs. high 13.7 ± 7.9 RFUs) (*p* = 0.285) and 14 (low 25.6 ± 11.9 vs. high 11.9 ± 10.0 RFUs) (*p* = 0.111) using either Percoll thawing medium. 

## 3. Discussion

It is common methodology in PHH isolations to use a Percoll density gradient to purify cells, which can lead to increased cell yield, viability, and performance [15,18]. Percoll gradients are also used to separate the different cell types in the liver, including Kupffer cells, stellate cells, and liver sinusoidal endothelial cells based on the different densities of Percoll after centrifugation [19]. The effect of using a Percoll gradient to isolate hepatocytes has been examined [20]; however, the outcome of using a Percoll gradient to enrich cryopreserved diseased PHHs post-thaw has not been as thoroughly assessed [21,22,23]. This current study was performed using PHHs isolated from diseased and normal donors to compare phenotype and functionality after being enriched in either a low Percoll or high Percoll thawing medium. 

Patients with T2DM show an increased prevalence of NAFLD [24,25]. The influence of T2DM on NAFLD and vice versa with regard to disease progression is still unclear. However, the pathogenic mechanism between the two most likely interact [26]. Although the T2DM donor lots tested were not classified as diseased, they potentially could serve to demonstrate how each impacts the other, specifically when examining fibrosis. After the T2DM donor PHHs were enriched in the different thawing media, notable differences in morphology, functionality, and marker expression were observed. PHHs enriched in high Percoll appeared to have a more diseased morphology compared to PHHs enriched in low Percoll for donor lot 2113766. Although these high Percoll-enriched PHHs had greater albumin levels and CYP3A4 activity, fibrotic marker expression of CK18 and TGF-β was elevated when the fluorescent signal was quantitated in this donor. However, in donor lot 2118545, these morphology and functional differences were reversed. Despite a difference in the albumin level between the low and high Percoll-enriched PHHs, the high Percoll-enriched PHHs had less fibrotic marker expression and lipogenesis in this donor. 

Upon examination of medical history, donor 2113766 had coronary artery disease (CAD) and a history of hypertension. Studies conducted by Vega et al. found that patients with liver fibrosis or CAD more frequently had T2DM, making it a confounding factor [27]. Researchers have suggested a group of individuals that have been diagnosed with early-stage liver fibrosis (F1) plus T2DM have an increased risk of more severe liver disease [27]. These individuals have been designated as rapid progressors [28]. Donor 2113766 may have been a rapid progressor with clear indicators of fibrosis upon histological examination and PHH characterization, but this was not suspected due to their continued normal liver function. It has been noted that liver enzymes are not a sensitive marker of NAFLD [25,29]. Completely normal liver enzymes were measured from some patients with the most severe form of liver disease [30]. This study observed the range of fibrosis across individuals with normal enzyme values, designating these patients as normal yet in reality were diseased. 

In addition to testing T2DM donor lots, diseased lots with NAS scores ≥4 were examined. Two of these donor lots, 1811122 and 16096, had a NAS score of 5 and 4, which includes their steatosis score. Both lots showed similar diseased morphology and had no significant differences in attachment after enrichment in either low or high Percoll. However, when Nile Red fluorescence was quantified, there were significant differences between the low and high Percoll-enriched PHHs. Those PHHs enriched in the low Percoll had significantly more lipid accumulation compared to the PHHs enriched in the high Percoll. This occurrence in the diseased PHHs has been seen during hepatocyte isolations from mice with fatty liver [31]. When a low Percoll gradient (25%) was used during the isolation, there were higher numbers of lipid-filled hepatocytes present compared to the isolation that used a high Percoll gradient (90%) [32]. A similar occurrence may also have happened with the use of different Percoll percentages post thaw. 

The expression of the gluconeogenesis genes, *G6PC* and *PCK1*, was examined in these donors. Both diseased donor lots had significantly lower *G6PC* gene expression after PHHs were enriched in low Percoll. Although a similar result was seen for both diseased donor lots for *PCK1* gene expression, only donor lot 16096 had significantly lower expression when the PHHs were enriched in low Percoll. A study by Ye et al. showed that *PCK1* is downregulated in NASH patients [32]. This study also found that reduction of *PCK1* stimulated inflammation and fibrogenesis in MAFLD mice [33]. The results found in our gene expression study suggest that donor lot 1811122 may have a homogenous population of diseased PHHs with similar characteristics that cannot be enriched further with the use of different Percoll densities. However, donor lot 16096 may have a heterogenous population of diseased PHHs that can be enriched from the other population by using specific Percoll densities. 

Further evidence of this is demonstrated when examining functionality differences after PHHs were enriched in either low or high Percoll. Only diseased donor lot 16096 showed a significant decrease in albumin and urea levels after enrichment in low Percoll compared to those PHHs enriched in high Percoll. There were no significant differences in albumin and urea levels in PHHs after enrichment in either low or high Percoll for diseased donor lot 1811122. Baseline CYP3A4 activity was significantly higher in the PHHs enriched in the high Percoll in both diseased lots and the normal lot. Overall, donor lot 1811122 had the lowest urea level and CYP activity compared to the other two lots. This donor had a hepatocyte ballooning score of 2, higher than the score of 1 for donor 16096. One study found that urea synthesis and CYP3A4 activity, but not albumin secretion, were reduced in induced human ballooned hepatocytes in a 3D model [33]. It may be due to the increased steatosis and hepatocyte ballooning scores that a homogenous cell population was present regardless of the amount of Percoll in the thawing medium for donor lot 1811122. 

Donor lots 2118143 and 2116167, representing high fibrosis, were the final lots used to determine the outcome on PHHs after using either low or high Percoll post-thaw. Differences in morphology, attachment, lipogenesis, functionality, and fibrosis were seen in PHHs enriched in low Percoll when compared to the PHHs enriched in high Percoll. Because both donor lots had a steatosis score of 1, significantly higher values of Nile Red fluorescence were quantitated in the low Percoll-enriched PHHs. A decline in serum albumin concentration has been associated with steatosis in NAFLD patients [34]. Studies have also shown urea cycle dysregulation in NASH patients [35]. Mitochondrial urea cycle enzymes were reduced, leading to hyperammonia and reduced hepatic capacity to synthesize urea through the urea cycle [35,36,37]. Because lipogenesis was lowered in the PHHs enriched in the high Percoll, there was no decrease in albumin and urea function. Expression of the fibrosis marker CK18 was significantly higher in the PHHs enriched using the low Percoll on both days 7 and 14. It may be that the enriched PHHs from these donors have varying levels of fibrosis that cannot be determined until the cells are cultured. 

Notably, only diseased fibrotic donor lot 2116167 (NAS score of 4) had significantly higher CD68 expression on day 14 in the low Percoll-enriched PHHs compared to the high Percoll-enriched PHHs. This lot also had the highest fluorescent values. It is interesting to note that fibrotic lot 2118143 (NAS score of 3) did not have significant differences in fluorescent values between the two different Percoll-enriched PHHs. The higher level of CD68 marker expression in donor lot 2116167 may be attributable to hepatocyte ballooning, which donor lot 2118143 did not have. CD68-positive expressing cells are commonly designated as pro-inflammatory macrophages because they secrete pro-inflammatory cytokines [38]. It has been shown that hepatocyte ballooning is associated with higher inflammation [39] and indicates an increased risk of more severe liver disease outcomes. 

In addition to CD68 expression, both fibrotic lots expressed CD163. No significant differences were seen between PHHs enriched in low versus high Percoll. CD163-positive cells are referred to as anti-inflammatory macrophages because they secrete anti-inflammatory cytokines [38] and have been shown to be involved in tissue repair, which may be pro-fibrotic [40,41]. Neither donor lot displayed significant differences in CD163 expression levels between PHHs enriched in the low and high Percoll, possibly because of the dual anti-inflammatory/tissue repair functionality of CD163-positive cells [42].

In conclusion, PHHs were able to be separated into distinct populations using low and high Percoll after thawing. These distinctions were based on diseased characteristics including steatosis, inflammation, and fibrosis. Variations in functionality and marker expression were also seen from these divergent populations. Overall, the use of Percoll to enrich diseased PHHs allows retention of a diseased phenotype with changes in attachment, function, and lipogenesis in the TruVivo^®^ system. In addition, alterations in fibrotic and macrophage marker expression can also be determined through using different Percoll-based thawing media, enabling researchers to select the best choice for post-thaw processing based on the desired endpoint measures.

## 4. Materials and Methods

### 4.1. TruVivo^®^ System

TruVivo^®^ was set up as previously described, including the media, cell types, and expected functional performance [12]. PHHs were thawed in medium containing either low (diluted four-fold) or high (standard) Percoll. A total of 300,000 PHHs and 50,000 human FCs were seeded per well in a 24-well plate. All methods were performed in accordance with the guidelines and regulations of LifeNet Health’s ethics committee. Informed consent was obtained for all donor tissue for research purposes by LifeNet Health. PHHs were designated as normal or diseased based on a histopathologic assessment of the tissue of origin by a board-certified liver pathologist, using the standard NASH CRN scoring system [43]. Tissues with a NAS score of ≥4 were designated “Diseased”, while those with a NAS score of ≤3 were categorized as “Normal” (Table 1). 

### 4.2. Morphological Assessment 

To morphologically assess the cells, they were imaged on designated days using a BX41 microscope (Olympus, Tokyo, Japan) or Zeiss Observer.Z1 fluorescent microscope (Zeiss, Dublin, CA, USA). 

### 4.3. Calculating PHH Attachment 

The previously described method was used to determine PHH attachment [13]. 

### 4.4. Albumin and Urea Assays

Supernatant was collected on the indicated days for measurement of albumin and urea. Two wells or greater were designated for each condition unless otherwise stated. Samples were run in duplicate. The concentration of albumin was determined using an ELISA assay (Abcam, Cambridge, MA, USA) and performed according to the manufacturer’s instructions. Urea was measured by a colorimetric kit (Stanbio, Boerne, TX, USA) and performed according to the manufacturer’s instructions. 

### 4.5. Basal CYP3A4 Activity Assay

The P450-Glo Assay kit was used to detect baseline CYP3A4 enzyme activity (Promega, Madison, WI, USA). Three wells or greater were designated for each condition unless otherwise stated. Samples were run in duplicate. After 24 h, the medium was removed, and the cells were washed with DMEM (no phenol red) (Thermo Fisher, Waltham, MA, USA). Cyp-Luciferin-IPA stock was then added and incubated at 37 °C for 30 min. The supernatant was then collected, and the assay was performed according to the manufacturer’s instructions. Samples were run in duplicate. 

### 4.6. Gene Expression

Cells were lysed using RLT buffer (Qiagen, Germantown, MD, USA). RNA was then isolated using the RNeasy kit (Qiagen) as per the manufacturer’s instructions. cDNA was prepared using the PrimeScript RT reagent kit (Takara Bio, Shiga, Japan) in a 30 µL volume reaction containing 6 µL 5× PrimeScript RT Master Mix, 20 µL RNA, and 4 µL ddH_2_O. qRT-PCR reactions contained 10 µL QuantiNova 2× SYBR Green Master Mix (Qiagen), 2 µL ROX reference dye (1:10 dilution; Qiagen), 2 µL designated primer set (10 pM), 1 µL cDNA, and 5 µL ddH_2_O for a final volume of 20 µL. Primer sequences used for *GAPDH*, *G6PC*, and *PCK1* (Thermo Fisher) are shown in Table 2. PCR amplification was done on a QuantStudio^TM^ 7 Flex Real-time PCR System (Thermo Fisher) using the following program: Step 1: 02:00 min at 95 °C; Step 2: 00:05 s at 95 °C; Step 3: 00:10 s at 60 °C. Steps 2 and 3 were repeated for 40 cycles. Data were analyzed with QuantStudio^TM^ 7 Flex Real-Time PCR System software v1.6 (Thermo Fisher) and Microsoft Excel. Gene expression was normalized to the housekeeping gene *GAPDH* and analyzed using the 2^−ΔΔCT^ method [44,45,46,47]. It is acknowledged that the use of *GAPDH* as the housekeeping gene could lead to the exclusion of certain data sets. Data are presented as fold change relative to PHHs enriched in high Percoll. 

### 4.7. Immunofluorescence and Quantitation

Cells were fixed with a fixation solution (eBioscience, San Diego, CA, USA) for 30 min (mins) at 4 °C. They were then washed two times with 1× permeabilization (eBioscience), and primary antibody was added at 4 °C overnight. The following antibodies were used: (1) CD68 at 1:100 (Abcam, ab955); (2) CD163 at 1:100 (Abcam, ab87099); and (3) CK18 at 1:1000 (Abcam, ab24561); and (4) TGF-β at 1:100 (Abcam, ab92486). Cells were washed twice, and secondary goat anti-mouse IgG Alexa Fluor 488 conjugated antibody (Thermo Fisher) or secondary goat anti-rabbit IgG Alexa Fluor 555 conjugated antibody (Thermo Fisher) was added at a 1:500 dilution for 30 min at 4 °C. Cells were then washed twice, and Fluoromount-G mounting medium with DAPI (Invitrogen, Waltham, MA, USA) was added for 20 min at room temperature. Images were taken using a Zeiss Observer.Z1 fluorescent microscope.

To quantitate the signal, five images were taken from a pre-determined location for each sample well [14]. Each well was focused in the 5× objective, followed by capturing images at the 10× objective. Exposure for DAPI, dsRed, and GFP was set at 150 ms, 1000 ms, and 2000 ms, respectively. Images were processed in ZEN 2012 software (blue edition) to remove fluorescent background by adjusting image grey values. All fluorescent channels were set using the same grey values determined for their specific filter. Individual and merged images were exported and opened in ImageJ. Individual channels were measured for fluorescent intensity using the “measure” function in ImageJ set to record integrated density. Quantitation of signal is represented in relative fluorescent units (RFUs). 

### 4.8. Nile Red Staining

Cells were washed with 1× DPBS (-Ca^++^/-Mg^++^) (Thermo Fisher) three times, and then Nile Red (Abcam) was added at a 1:500 dilution. After 15 min at 37 °C, cells were washed twice with 1× DPBS (-Ca^++^/-Mg^++^) and then imaged on an EVOS FL cell imaging system (Thermo Fisher) using a 10× or 20× objective. Nile Red staining was quantified by determining the densitometric fluorescence value (red channel) using ImageJ. Values were normalized to the determined number of attached PHHs as described above. 

### 4.9. Statistical Analysis

Images shown are from representative donor lots. For albumin, urea, and CYP3A4 measurements, values were normalized to the determined number of attached PHHs as described above. Significance was calculated in MiniTab (State College, PA, USA) using either Student’s *t*-test or one-way ANOVA with Tukey post hoc testing to determine statistical significance with 95% confidence and * *p* < 0.05 for statistical significance. The Student’s *t*-test was used to calculate significance when comparing two groups, while the ANOVA with Tukey post hoc testing was used when comparing three groups or more.

## 5. Conclusions

In conclusion, enrichment of distinct populations of PHHs was determined by using low and high Percoll after thawing. In addition to functional differences, there were differences in steatosis, inflammation, and fibrosis that were evident in each population. Because of these alterations, a selected diseased phenotype was maintained, which allows users to choose the optimal thawing medium for post-thaw processing depending on a desired endpoint measure. 

## Figures and Tables

**Table 1:** Donor characteristics.

Donor	Age	Sex	Race	BMI	NAS Score	Steatosis Score	Lobular Inflammation Score	Hepatocyte Ballooning Score	Fibrosis Stage	Health Category
16117	41	F	Caucasian	30	0	0	0	0	0	Normal
2118143	44	M	Caucasian	30.1	3	1	2	0	2	Normal + Fibrosis
2116167	51	M	Caucasian	29.8	4	1	1	2	1	Disease
16096	73	F	Caucasian	30	4	2	1	1	0	Disease
1811122	28	F	Caucasian	34	5	3	0	2	1A, Focal	Disease
2113766	56	M	Hispanic	23.9	1	0	1	0	2	T2DM
2118545	59	M	Caucasian	28.6	2	1	0	1	0	T2DM

**Table 2:** Primer sequences.

Primer Name	Forward Sequence (5′-3′)	Reverse Sequence (5′-3′)
*GAPDH*	GGTCACCAGGGCTGCTTTTA	GGATCTCGCTCCTGGAAGATG
*G6PC*	TCATCTTGGTGTCCGTGATCG	TTTATCAGGGGCACGGAAGTG
*PCK1*	ACTCGAGGTTCTGCACCCCT	AGGCAGCATCAATGATGGG

## Data Availability

The datasets generated during and/or analyzed during the current study are available from the corresponding author on reasonable request.
